# Symptoms of Depression and Anxiety among Myopes: A Systematic Review and Meta-Analysis

**DOI:** 10.22599/bioj.500

**Published:** 2026-02-11

**Authors:** Samuel Kyei, Randy Asiamah, Gideon Owusu, Emmanuel Ekow Ampiah

**Affiliations:** 1 Department of Ophthalmic Science, School of Optometry and Vision Science, College of Health and Allied Sciences, University of Cape Coast, Cape Coast, Ghana; 2 Biomedical and Clinical Research Centre, College of Health and Allied Sciences, University of Cape Coast, Cape Coast, Ghana; 3 School of Optometry and Vision Science, College of Health and Allied Sciences, University of Cape Coast, Cape Coast, Ghana; 4 Department of Vision Science, School of Optometry and Vision Science, College of Health and Allied Sciences, University of Cape Coast, Cape Coast, Ghana

**Keywords:** myopia, anxiety, depression, mental health, emmetropia

## Abstract

**Objectives:**

To assess the prevalence of symptoms of depression and anxiety, as well as associated factors in myopes.

**Methods:**

Relevant studies were sourced from PubMed, Scopus, and Web of Science. A meta-analysis was conducted to determine the prevalence of symptoms of depression and anxiety in myopes. Odds ratios (OR) with corresponding 95% confidence intervals (CI) were used to compare psychological symptoms between myopic and emmetropic groups.

**Results:**

A total of six studies assessing the symptoms of depression and anxiety in myopes were included. Prevalence of symptoms of depression and anxiety in myopes are 22.99% (95% CI 16.81% to 30.61%; *I*^2^ = 96.6%) and 26.81% (95% CI 15.62% to 42.01%; *I*^2^ = 98.4%), respectively. Myopes are approximately 46% more likely to suffer from symptoms of depression [OR: 1.46 (95% CI 0.98 to 2.19), *I*^2^ = 72.4%] and are 65% more likely to suffer from symptoms of anxiety [OR: 1.65 (95% CI 1.10 to 2.49), *I*^2^ = 70.4%], as compared to emmetropes. Myopes aged less than 40 years are about 12% more likely to suffer from symptoms of depression [OR: 1.12 (95% CI 0.83 to 1.50), *I*^2^ = 1.5%] and 26% more likely to suffer from symptoms of anxiety [OR: 1.26 (95% CI 1.11 to 1.43), *I*^2^ = 72.5%] than age-matched emmetropes. Myopes aged 40 years and older are almost twice as likely to suffer from symptoms of depression than age-matched emmetropes [OR: 2.02 (95% CI 1.61 to 2.51), *I*^2^ = 0.0%].

**Conclusion:**

Integrated eye care approaches are advocated for and should consider the psychological impact of visual impairment. As myopia prevalence rises globally, understanding and mitigating its mental health effects will be crucial for public health.

## Introduction

Myopia, or near-sightedness, is a significant global public health concern that has reached epidemic proportions in recent decades. It is characterised by the inability to see distant objects clearly due to elongation of the eyeball. It is the most common refractive error worldwide, and its prevalence has been rising sharply, particularly among children and adolescents (Surico *et al*., 2024; Liang *et al*., 2025). Recent estimates indicate that approximately 30% of the global population was myopic in 2020 but projections suggest this will increase to nearly 50% by 2050, affecting over 4.8 billion people globally (Liang *et al*., 2025; Xiang and Zou, 2020). The burden of myopia is particularly pronounced in East Asia, where up to 90% of school-leavers in countries like China, Japan and Singapore are affected (Rudnicka *et al*., 2016; Grzybowski *et al*., 2020). This alarming trend has been attributed to an interplay of genetic predisposition and environmental factors such as increased near work (e.g., reading and screen use) and reduced time spent outdoors (Foster and Jiang, 2014; Landreneau, Hesemann and Cardonell, 2021). The epidemiology of myopia reveals striking regional and demographic differences. Urbanisation and socioeconomic status also play a critical role; children living in urban areas are more likely to develop myopia compared to their rural counterparts due to lifestyle differences, including reduced exposure to natural light (Grzybowski *et al*., 2020). Furthermore, high myopia (defined as a refractive error of –6.00 diopters or worse) is on the rise globally and is associated with severe complications such as retinal detachment, glaucoma, and myopic macular degeneration (Landreneau, Hesemann and Cardonell, 2021; Tomiyama, 2024). These complications underscore the need for early detection and effective management strategies.

Beyond its physical implications, myopia is increasingly recognised for its psychological impact on affected individuals (Osuagwu *et al*., 2023; Lu *et al*., 2024). Adolescents with myopia often report higher levels of anxiety and depression compared to their peers with normal vision (Rudnicka *et al*., 2016; Foster and Jiang, 2014; Huang *et al*., 2022; Nitzan, Shmueli and Safir, 2024). This may be attributed to several factors, including the stigma associated with wearing glasses, reduced participation in sports or outdoor activities, and concerns about future vision loss. Studies have also shown that children with moderate-to-severe myopia score lower on vision-related quality-of-life measures, which are closely linked to mental health outcomes (Zhang *et al*., 2024). However, findings regarding the relationship between myopia and depression remain inconsistent. While some studies report significant associations between severe myopia and depressive symptoms, others find no such correlation (Foster and Jiang, 2014; Li *et al*., 2023).

Despite these observations, the psychological consequences of myopia remain underexplored compared to its physical complications. Existing studies vary widely in methodology, population demographics and outcome measures, resulting in conflicting conclusions. Moreover, while interventions such as increased outdoor activities have shown promise in reducing the progression of myopia, their impact on alleviating psychological symptoms remains unclear (Liang *et al*., 2025; Xiong *et al*., 2017). This gap highlights the need for a comprehensive synthesis of evidence to better understand the interplay between myopia and mental health.

This systematic review and meta-analysis aim to assess the prevalence of symptoms of depression and anxiety, as well as associated factors in myopes. By consolidating findings from diverse studies across different populations and age groups, this review seeks to quantify these associations and identify research gaps that warrant further investigation. Ultimately, this work aims to provide valuable insights into the broader impact of myopia on mental health and inform strategies for early detection and holistic management.

## Methods

This systematic review and meta-analysis is registered in PROSPERO (CRD42025648360) and was conducted according to the Preferred Reporting Items for Systematic Reviews and Meta-Analyses (PRISMA) statement guidelines (Page *et al*., 2021).

### Eligibility criteria

The systematic review and meta-analysis include cross-sectional, case-control and cohort studies published since the first date of publication of each of the journals up to 2025. Studies that reported psychological symptoms such as anxiety, depression and sleep disorders in myopic individuals of all age groups, as assessed by any validated scale, were included. Diagnostic classifications of myopia as applied in the included studies were accepted, and the quantitative definitions proposed by Flitcroft (2018) were used in the creation of subgroups according to the severity of myopia (low myopia and high myopia). Low myopia includes spherical equivalent refractive errors of between ≤ –0.5D and > –6.00D when ocular accommodation is relaxed, and high myopia includes spherical equivalent refractive errors of ≤ –6.00D when ocular accommodation is relaxed.

Database searches were not limited to studies published in English. Primary studies that were not published in full in peer-reviewed journals were excluded from the analysis. Studies with unavailable full text and those that repeated data from the author’s previous publications were also excluded. Narrative reviews, discussion papers, non-research letters or editorials, case series, case reports and animal studies were excluded from the analysis. Primary studies with significant amounts of missing data were excluded from the analysis.

### Information sources

A systematic search was performed across three major databases: *Web of Science, PubMed*, and *Scopus*, covering the period from the dates of the journals’ inception to February 2025. Additionally, the reference lists of the selected articles were meticulously reviewed to identify any additional potentially pertinent studies. The search strategy included the terms: (‘prevalence’ OR ‘epidemiology’) AND (‘psychol* AND disorder’ OR ‘psychol* AND distress’ OR ‘emotion* AND disorder’ OR distress OR stress OR anxiety OR depression) AND (myop* OR ‘short AND sight*’ OR ‘near AND sight*’). A detailed outline of the search strategy used in each database is available in a supporting information file (Table S1). All identified records were imported into the systematic review management platform, Covidence (Veritas Health Innovation, www.covidence.org), for title and abstract, and full-text screening.

### Selection process

**Figure 1:**
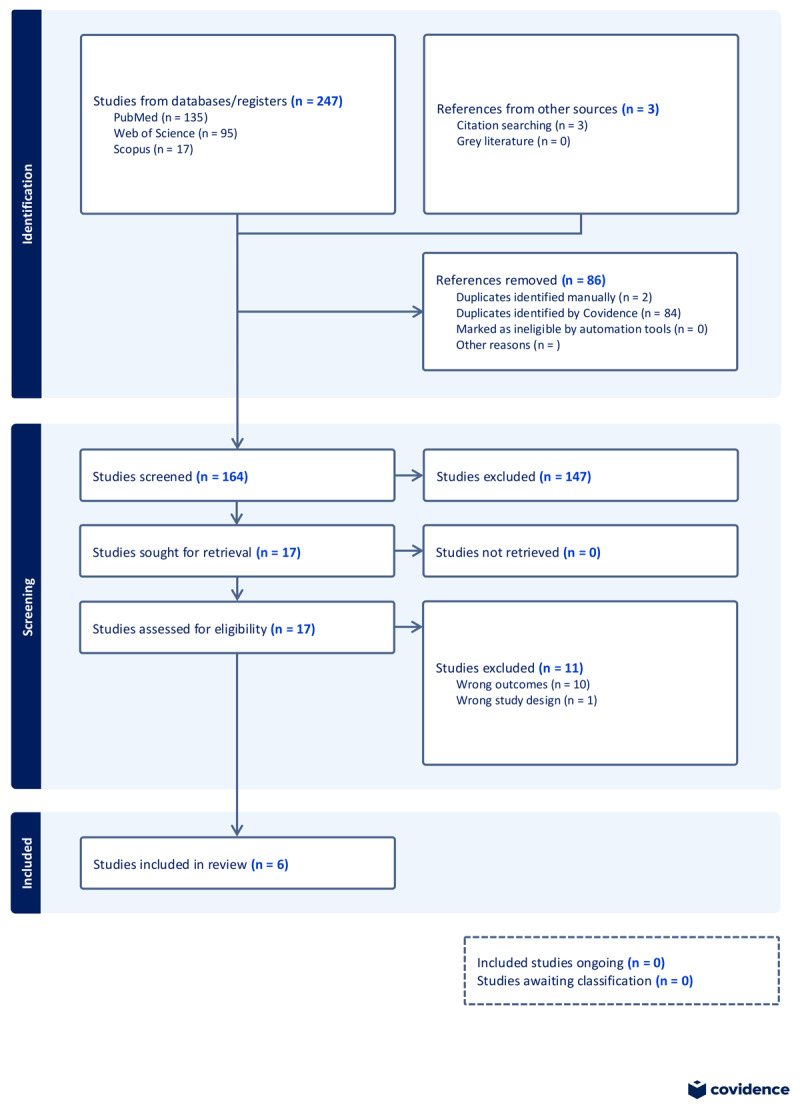
PRISMA flowchart.

Relevant studies retrieved from the database searches were screened by two independent reviewers (GO and EA), first by title and abstract and then by full text. Disagreements between the pair were resolved by consensus or consultation with the project PI (SK). The Preferred Reporting Items for Systematic Reviews and Meta-Analysis (PRISMA) flow diagram was employed to report the screening process (Figure 1).

### Data collection process

Two reviewers (RA and GO) extracted data from each eligible study using a standardised data extraction sheet and subsequently cross-checked the results. The data extracted included the first author’s name, year of study, country, myopia definition used, myopia severity, psychological symptom assessed, assessment tool for psychological symptoms, mean scores of psychological symptoms, number of myopes and number of myopes with psychological symptoms. Disagreements regarding the extracted data were resolved through discussion with a third reviewer (SK).

### Data synthesis and analysis

Study-specific estimates of the prevalence of psychological symptoms in myopes were pooled using the one-group meta-analysis in a random-effects model using the R (version 4.4.3) package meta. The likelihood of psychological symptoms occurring in the myopic groups, as compared to the emmetropic groups, was estimated using odds ratio (OR) and corresponding 95% confidence intervals (CI).

Heterogeneity was assessed using Cochran’s Q and quantified using *I*^2^, with heterogeneity estimates of 25%, 50% and 75% representing low, moderate and high heterogeneity, respectively. The output included a test of whether the intercept differed significantly from zero, and whether the other groups differed from the intercept.

A sensitivity analysis was conducted to ascertain the robustness of the pooled prevalence estimates through the implementation of a leave-one-out meta-analysis approach (Patsopoulos, Evangelou and Ioannidis, 2008). This approach entailed the assessment of the influence of a specific study on the between-study heterogeneity by the systematic exclusion of each study in succession (Patsopoulos, Evangelou and Ioannidis, 2008). Publication bias was not estimated, as the number of studies included in the meta-analysis was less than 10 (Egger *et al*., 1997). All *p*-values were two-sided, and those less than 0.05 were considered statistically significant.

### Study risk of bias assessment

The methodological qualities of the included studies were assessed according to the Newcastle-Ottawa Scale (NOS) (Lo, Mertz and Loeb, 2014) by two reviewers (SK and RA). Three evaluation criteria were considered: case selection, comparability, and exposure. Quality scores ranged from 0 to 9, and studies that scored above 6 were considered high quality. Scores 4–6, < 4 were considered intermediate and high risk, respectively.

## Results

**Figure 2:**
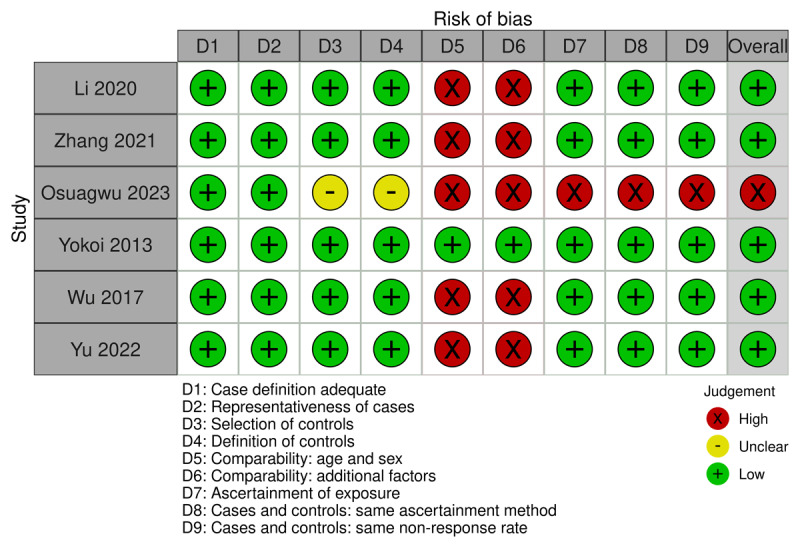
Risk of bias by domain and question for studies included in the meta-analysis. **SDS:** Self-Rating Depression Scale, **SAS:** Self-Rating Anxiety Scale, **BDI:** Beck Depression Inventory, **HADS-D:** Hospital Anxiety and Depression Scale – Depression Subscale, **HADS-A:** Hospital Anxiety and Depression Scale – Anxiety Subscale, **DASS-21:** Depression Anxiety Stress Scales – 21 Items, **PHQ-9:** Patient Health Questionnaire – 9 Items, **SE:** Spherical Equivalent (Li *et al*., 2023).

Database searches retrieved 297 potentially relevant articles, with three studies obtained from citation searches. After deduplication, 164 studies remained for title and abstract screening. Out of the 164 titles and abstracts screened, 17 studies were sought for full-text retrieval, and 11 were excluded due to inappropriate outcomes (10 studies) and wrong study design (one study). A total of six studies met the inclusion criteria and were included in the systematic review and meta-analysis. A summary of the risk of bias of the six studies, as assessed using the NOS, is provided in Figure 2.

### Study characteristics

The studies included in this review were published between 2013 and 2023. A total of five studies were conducted among Asian populations, while one study was conducted among an African population. The most commonly assessed psychological symptoms among myopes were found to be anxiety and depression. While five of the studies assessed for depression among myopes, four of the studies assessed for anxiety among myopes. The included studies involved a total of 12,756 individuals. Of these, depression was assessed in 2,932 myopes (680 myopes with depression) and 3,792 emmetropes (311 emmetropes with depression). Also, anxiety was assessed for 6,640 myopes (2179 myopes with anxiety) and 1,536 emmetropes (442 emmetropes with anxiety). Again, depression was assessed in 3,079 males and 3,645 females, while anxiety was assessed in 2,946 males and 5,230 females. Participants with low vision or amblyopia were not included in the studies. The most used tool in assessing depression was the Zung Self-Rating Depression Scale (SDS) (Zhang *et al*., 2021; Li *et al*., 2023) and the most used tool in assessing anxiety was the Zung Self-Rating Anxiety Scale (Zhang *et al*., 2021; Li *et al*., 2023) (Table 1).

**Table 1:** Characteristics of included studies.


AUTHOR	COUNTRY	AGE GROUP	MYOPIA DEFINITION USED	REFRACTIVE MEASUREMENT USED	PSYCHOLOGICAL SYMPTOMS ASSESSED	ASSESSMENT TOOLS USED

Yokoi 2014 (Yokoi *et al*., 2014)	Japan	40 years and above (60+ years)	AL greater or equal to 26.50mm	–	Depression, Anxiety	HADS-D (Depression), HADS-A (Anxiety)

Wu 2017 (Wu *et al*., 2017)	China	40 years and above (60+ years)	SE of –0.5D or less. High myopia (SE >6.00D).	Non-cycloplegic autorefraction	Depression	PHQ-9 (Depression)

Li 2023 (Li *et al*., 2023)	China	Less than 40 years (Mean: 15 years, Range: 14.1 to 17.5)	SE of –0.5D or less. Mild myopia (SE <3.00D), moderate myopia (SE 3.00 to 6.00D), and severe myopia (SE >6.00D).	Cycloplegic autorefraction	Depression, Anxiety	SDS (Depression), SAS (Anxiety)

Zhang 2021 (Zhang *et al*., 2021)	China	Less than 40 years (Mean: 18.2 ± 0.7 years, Range: 15 to 23 years)	SE of –0.5D or less. Mild myopia (SE <3.00D), moderate myopia (SE 3.00 to 6.00D), and severe myopia (SE >6.00D).	Non-cycloplegic autorefraction	Depression, Anxiety	SDS (Depression), SAS (Anxiety)

Yu 2022 (Yu *et al.*, 2022)	China	Less than 40 years (Mean: 19.82 ± 1.43 years, Range: 15 to 25 years)	–	–	Anxiety	DASS-21 (Anxiety)

Osuagwu 2023 (Osuagwu *et al*., 2023)	Nigeria	All (16+ years)	SE of –5.00 or less	–	Depression	BDI (Depression)


### Symptoms of depression

**Figure 3:**
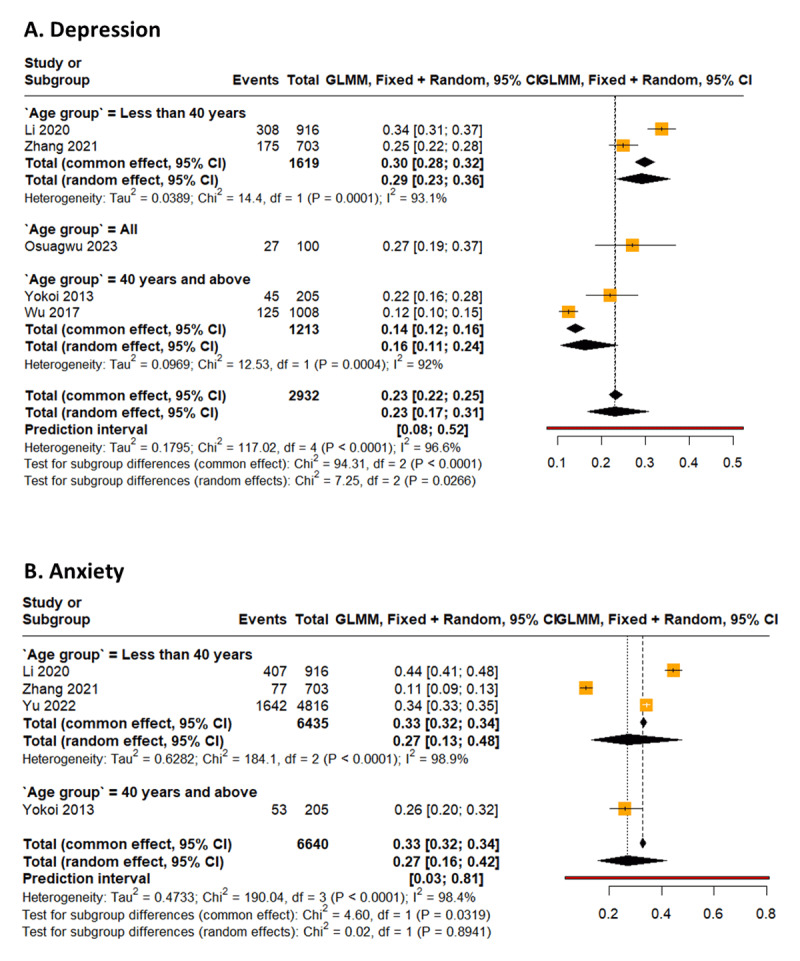
Forest plot of the prevalence of psychological symptoms (anxiety and depression) among myopes.

**Figure 4:**
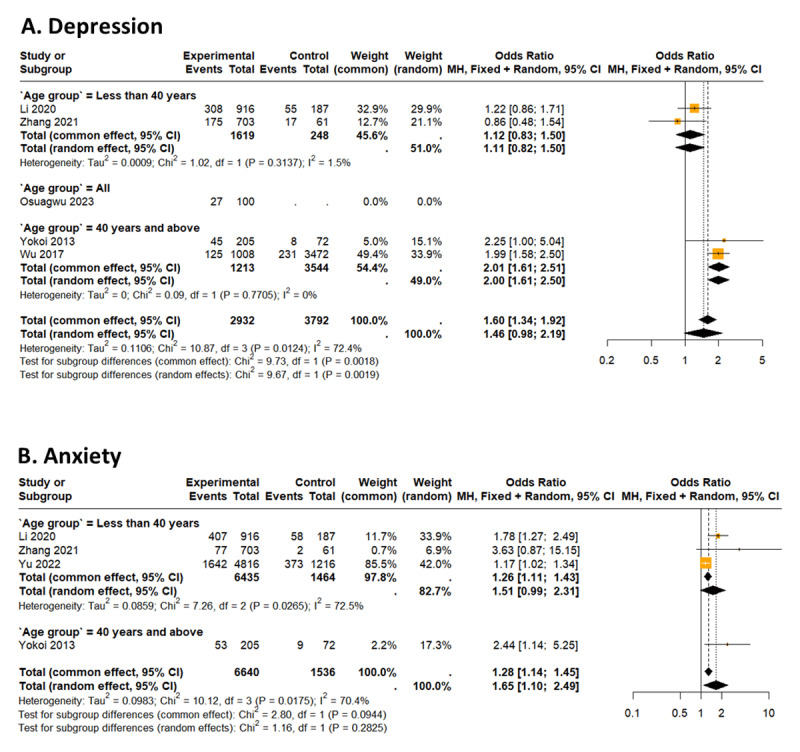
Forest plot of the association between psychological symptoms (anxiety and depression) and myopia.

Prevalence of symptoms of depression in myopes is 22.99% (95% CI 16.81% to 30.61%; PI: 7.58 to 52.28, *I*^2^ = 96.6%) (Li *et al*., 2023; Zhang *et al*., 2021; Osuagwu *et al*., 2023; Yokoi *et al*., 2014; Wu *et al*., 2017) (Figure 3). Moreover, myopes are approximately 46% more likely to be depressed than emmetropes [OR: 1.46 (95% CI 0.98 to 2.19), *I*^2^ = 72.4%] (Li *et al*., 2023; Zhang *et al*., 2021; Osuagwu *et al*., 2023; Yokoi *et al*., 2014; Wu *et al*., 2017) (Figure 4).

The prevalence of symptoms of depression in myopes aged less than 40 years is 29.83% (Li *et al*., 2023; Zhang *et al*., 2021) (95% CI 27.65% to 32.11%, *I*^2^ = 93.1%). Also, the prevalence of depression in myopes aged 40 years and older is 14.01% (95% CI 12.17% to 16.08%, *I*^2^ = 92.0%) (Yokoi *et al*., 2014; Wu *et al*., 2017) (Figure 3).

Myopes aged less than 40 years are about 12% more likely to be depressed than emmetropes within the same age group (Li *et al*., 2023; Zhang *et al*., 2021) [OR: 1.12 (95% CI 0.83 to 1.50), *I*^2^ = 1.5%]. Again, myopes aged 40 years and older are almost twice as likely to be depressed than emmetropes also aged 40 years and over (Yokoi *et al*., 2014; Wu *et al*., 2017) [OR: 2.02 (95% CI 1.61 to 2.51), *I*^2^ = 0.0%] (Figure 4).

### Symptoms of anxiety

Prevalence of symptoms of anxiety in myopes is 26.81% (95% CI 15.62% to 42.01%; PI: 3.05 to 80.99, *I*^2^ = 98.4%) (Li *et al*., 2023; Zhang *et al*., 2021; Yu *et al*., 2022; Yokoi *et al*., 2014) (Figure 3). Myopes are about 65% more likely to suffer from anxiety, as compared to emmetropes [OR: 1.65 (95% CI 1.10 to 2.49), *I*^2^ = 70.4%] (Li *et al*., 2023; Zhang *et al*., 2021; Yu *et al*., 2022; Yokoi *et al*., 2014) (Figure 4).

The prevalence of anxiety among myopes aged less than 40 years is 27.12% (95% CI 13.12% to 47.82%, *I*^2^ = 98.9%) (Li *et al*., 2023; Zhang *et al*., 2021; Yu *et al*., 2022) (Figure 3). Also, myopes aged less than 40 years are about 26% more likely to suffer from anxiety than emmetropes within the same age group [OR: 1.26 (95% CI 1.11 to 1.43), *I*^2^ = 72.5%] (Li *et al*., 2023; Zhang *et al.*, 2021; Yu *et al*., 2022) (Figure 4).

The results of the sensitivity analysis of the depression and anxiety subgroups indicated that the exclusion of any single study did not result in a significant alteration to the pooled prevalence estimates (Figure S1).

## Discussion

This systematic review and meta-analysis reveals a significant psychological burden associated with myopia, demonstrating that individuals with this refractive error experience higher rates of depression and anxiety compared to those with normal vision. The pooled global prevalence estimates indicate that nearly one in four myopes (22.99%) experiences depression, while over one in four (26.81%) report anxiety symptoms. These figures translate to a 46% increased likelihood of depression and a 65% greater risk of anxiety among myopes relative to emmetropes. These findings align with and extend previous research linking visual impairment to poorer mental health outcomes (Crews *et al*., 2014), while providing novel insights into the specific psychological challenges faced by myopic individuals across different life stages.

A particularly striking finding emerges when examining age-related patterns in psychological symptoms. Younger myopes under 40 years show a depression prevalence of 29.83%, markedly higher than the 14.01% observed in older adults. However, the clinical significance of these numbers becomes clearer when considering the relative risks: while younger myopes are only marginally more likely to be depressed than their peers with normal vision (OR 1.12), older myopes face twice the risk of depression compared to age-matched emmetropes (OR 2.02). This pattern suggests that while depression may be more commonly reported among younger populations (Theron *et al*., 2025), generally, myopia confers a particularly heavy psychological burden in later life (Keyes, Kreski and Patrick, 2024). The cumulative effects of decades of managing visual impairment, coupled with concerns about progressive vision loss and functional limitations, likely contribute to this heightened vulnerability in older adults (Long *et al*., 2020; Yang *et al*., 2024).

Anxiety presents a different temporal pattern, maintaining consistently elevated rates across all age groups. The 27.12% prevalence among younger myopes, coupled with their 26% increased risk compared to emmetropes, underscores anxiety’s role as an immediate psychological associated factor to myopia. Unlike depression, anxiety levels remain persistently high throughout the lifespan, reflecting the ongoing daily challenges posed by refractive error, from academic pressures in youth to occupational demands in adulthood (Lenze and Wetherell, 2011). The immediate frustrations of blurred vision, social discomfort from corrective lenses, and constant awareness of visual limitations appear to sustain anxiety levels regardless of age (Hovenkamp-Hermelink *et al*., 2021).

Several mechanisms may underlie these psychological associations. Functionally, uncorrected refractive errors can impair performance in education, employment and leisure activities, potentially leading to frustration and reduced self-worth (Hopkins *et al*., 2020; Olatunji *et al*., 2019). Psychologically, the visible need for corrective lenses may affect self-image, particularly during adolescence when appearance concerns peak (Dudovitz *et al*., 2016). Biologically, emerging research suggests potential shared pathways between myopia development and mood regulation, including dopaminergic system involvement (Zhou *et al*., 2017; Yang *et al*., 2022). Furthermore, the very behaviours associated with myopia progression—excessive near work, limited outdoor activity, and prolonged screen time—are independently linked to poorer mental health outcomes, creating a potential vicious cycle (Dutheil *et al*., 2023; Huang, Chang and Wu, 2015; Sherwin *et al*., 2012).

The clinical implications of these findings are substantial. Eye care professionals should be alert to the psychological dimensions of refractive error, particularly when managing high myopia cases or younger patients. Routine vision assessments could incorporate brief mental health screenings, especially for patients reporting significant visual difficulties or compliance challenges with optical correction. For older myopes, who show particularly elevated depression risks, regular monitoring for mood symptoms is warranted.

From a public health perspective, interventions promoting outdoor activity could yield dual benefits—potentially slowing myopia progression while simultaneously supporting mental wellbeing through increased light exposure and physical activity (Rütten *et al*., 2017; Gormley *et al*., 2022; Manferdelli, La Torre and Codella, 2019). Educational initiatives normalising spectacle use and contact lens wear might help reduce the social stigma that contributes to anxiety, particularly among adolescents (Sadiq, Ayub and Ali, 2023). Workplace accommodations for visually demanding tasks could alleviate some of the occupational stressors faced by myopic adults (Basu and Sambath Rani, 2023).

Several limitations must be considered when interpreting these findings. The predominance of studies from Asian populations (five of six included studies) raises questions about generalisability to other population groups. The reliance on self-report measures for psychological symptoms, while practical, may be subject to reporting biases. The cross-sectional nature of most included studies prevents definitive conclusions about causality—we cannot determine whether myopia predisposes to psychological distress, whether mental health issues contribute to myopia development, or whether shared underlying factors drive both conditions. While most of the studies included in the meta-analysis were judged to have low risks of bias, as assessed using the NOS, most of the studies had quality concerns regarding comparability. Most of the studies lacked an explicit statement that the exposures of interest were adjusted for confounders such as age and sex, thus warranting the quality concern. The lack of adjustment for possible confounders could have resulted in spurious associations between myopia and the occurrence of psychological symptoms. The study by Osuagwu *et al*. (2023) was judged to have a high risk of bias as it did not assess psychological symptoms among a comparator group (emmetropes) (Osuagwu *et al*., 2023). The limited number of studies precluded a meta-regression analysis to investigate how various confounding factors, such as personal or environmental circumstances, influence depression and anxiety. This clearly indicates a gap in research that requires attention in future studies. Also, the limited number of studies precluded subgroup analysis by degree of myopia, which could have offered valuable insights into how the degree of myopia influences the association between the psychological symptoms and myopia. Another significant limitation of this meta-analysis stems from the heterogeneity in instruments used to assess psychological symptoms across included studies. Depression was assessed using five distinct scales (SDS, PHQ-9, BDI, HADS-D, DASS-21), while anxiety was measured with four different tools (SAS, HADS-A, DASS-21). This methodological diversity precluded precise quantification of estimates as clinical cutoffs for cases differed substantially across instruments (for example, SDS ≥ 50 against PHQ-9 ≥ 10 for depression). Also, tools like PHQ-9 were developed in Western populations, potentially misclassifying symptoms in Asian/African cohorts. Considering the identified limitations and potential sources of bias, it is imperative to exercise caution in interpreting the findings, considering the potential sources of bias that could have influenced the outcomes, and considering the exceptionally wide prediction intervals corresponding to the pooled estimates.

Future research should prioritise longitudinal designs that track refractive error and psychological symptoms over time, helping clarify temporal relationships. Studies incorporating more diverse populations would enhance the generalisability of findings. More sophisticated measurement approaches, including objective visual function assessments alongside confirmatory diagnosis by a clinical psychologist, could provide deeper insights into the mechanisms linking vision and psychological wellbeing.

## Conclusion

In conclusion, this systematic review establishes myopia as more than just a refractive error; it represents a significant risk factor for psychological distress across the lifespan. The distinct patterns observed for depression and anxiety suggest different underlying mechanisms and intervention opportunities. These findings advocate for more integrated approaches to eye care that recognise and address the psychological dimensions of visual impairment. As myopia prevalence continues to rise globally, understanding and mitigating its mental health impacts by implementing myopia interventions to potentially mitigate depressive symptoms, will become increasingly important for both individual wellbeing and public health.

## Data Accessibility Statement

Supporting data are available within the article.

## Additional File

The additional file for this article can be found as follows:

10.22599/bioj.500.s1 Supplementary Files. Table S1 and Figure S1.
