# Supplementary material for: Symptoms of Depression and Anxiety among Myopes: A Systematic Review and Meta-Analysis
Source: Br Ir Orthopt J. 2026 Feb 11;22(1):46–56. doi: 10.22599/bioj.500 (PMC12904118; doi:10.22599/bioj.500)
Supplement: Supplementary Files. — Table S1 and Figure S1. [file bioj-22-1-500-s1.zip › bioj-500_asiamah-s1/Table+S1.docx]

**PUBMED – Date: 19^th^ March, 2025**

| Search number | Query | Results |
| --- | --- | --- |
| 4 | ((((prevalence[Title/Abstract]) OR (epidemiology[Title/Abstract])) OR (prevalence[MeSH Terms])) AND ((((((((psychol*[Title/Abstract]) OR (distress[Title/Abstract])) OR (stress[Title/Abstract])) OR (anxiety[Title/Abstract])) OR (depression[Title/Abstract])) OR (depress*[Title/Abstract])) OR (mental health[Title/Abstract])) OR (emotional distress[Title/Abstract]))) AND (((myop*[Title/Abstract]) OR (short-sight*[Title/Abstract])) OR (near-sight*[Title/Abstract])) | 135 |
| 3 | ((myop*[Title/Abstract]) OR (short-sight*[Title/Abstract])) OR (near-sight*[Title/Abstract]) | 68,910 |
| 2 | (((((((psychol*[Title/Abstract]) OR (distress[Title/Abstract])) OR (stress[Title/Abstract])) OR (anxiety[Title/Abstract])) OR (depression[Title/Abstract])) OR (depress*[Title/Abstract])) OR (mental health[Title/Abstract])) OR (emotional distress[Title/Abstract]) | 2,304,954 |
| 1 | ((prevalence[Title/Abstract]) OR (epidemiology[Title/Abstract])) OR (prevalence[MeSH Terms]) | 1,213,193 |

**SCOPUS (17 results) – Date: 19^th^ March, 2025**

( ( ABS ( prevalence ) OR ABS ( epidemiology ) AND TITLE ( prevalence ) ) ) AND ( ( ABS ( psychol* AND disorder ) OR ABS ( psychol* AND distress ) OR ABS ( emotion* AND disorder ) OR ABS ( distress ) OR ABS ( stress ) OR ABS ( anxiety ) OR ABS ( depression ) ) ) AND ( ( TITLE-ABS-KEY ( myop* ) OR ABS ( short AND sight* ) OR ABS ( near AND sight* ) ) )

**WEB OF SCIENCE - Date: 19^th^ March, 2025**

| # | Search Query | Results |
| --- | --- | --- |
| 1 | ((TI=(prevalence)) OR AB=(prevalence)) OR AB=(epidemiology) | 1077076 |
| 2 | (((((((AB=(psychol* disorder)) OR AB=(psychol* distress)) OR AB=(emotion* disorder)) OR AB=(emotion* distress)) OR AB=(distress)) OR AB=(stress)) NOT AB=(anxiety)) OR AB=(depression) | 2623150 |
| 3 | AB=(myop*) | 55757 |
| 4 | #1 AND #2 AND #3 | 95 |
