# Supplementary figures and images for: Symptoms of Depression and Anxiety among Myopes: A Systematic Review and Meta-Analysis
Source: Br Ir Orthopt J. 2026 Feb 11;22(1):46–56. doi: 10.22599/bioj.500 (PMC12904118; doi:10.22599/bioj.500)

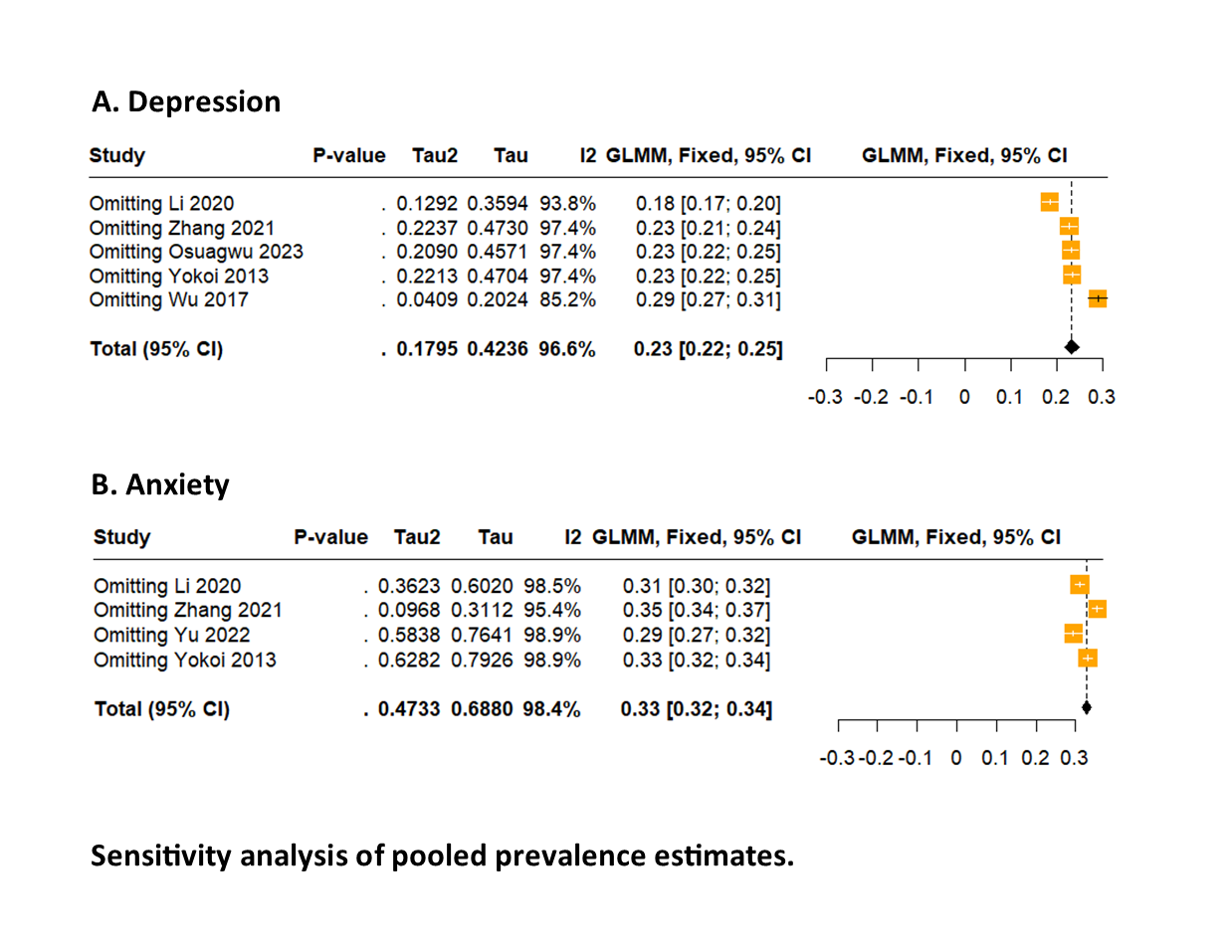

Supplement: Supplementary Files. — Table S1 and Figure S1. [file bioj-22-1-500-s1.zip › bioj-500_asiamah-s1/Figure+S1.tiff]
